# Chimira: analysis of small RNA sequencing data and microRNA modifications

**DOI:** 10.1093/bioinformatics/btv380

**Published:** 2015-06-20

**Authors:** Dimitrios M. Vitsios, Anton J. Enright

**Affiliations:** European Molecular Biology Laboratory—European Bioinformatics Institute, Wellcome Trust Genome Campus, Hinxton, Cambridge, CB10 1SD, UK

## Abstract

**Summary:** Chimira is a web-based system for microRNA (miRNA) analysis from small RNA-Seq data. Sequences are automatically cleaned, trimmed, size selected and mapped directly to miRNA hairpin sequences. This generates count-based miRNA expression data for subsequent statistical analysis. Moreover, it is capable of identifying epi-transcriptomic modifications in the input sequences. Supported modification types include multiple types of 3′-modifications (e.g. uridylation, adenylation), 5′-modifications and also internal modifications or variation (ADAR editing or single nucleotide polymorphisms). Besides cleaning and mapping of input sequences to miRNAs, Chimira provides a simple and intuitive set of tools for the analysis and interpretation of the results (see also Supplementary Material). These allow the visual study of the differential expression between two specific samples or sets of samples, the identification of the most highly expressed miRNAs within sample pairs (or sets of samples) and also the projection of the modification profile for specific miRNAs across all samples. Other tools have already been published in the past for various types of small RNA-Seq analysis, such as UEA workbench, seqBuster, MAGI, OASIS and CAP-miRSeq, CPSS for modifications identification. A comprehensive comparison of Chimira with each of these tools is provided in the Supplementary Material. Chimira outperforms all of these tools in total execution speed and aims to facilitate simple, fast and reliable analysis of small RNA-Seq data allowing also, for the first time, identification of global microRNA modification profiles in a simple intuitive interface.

**Availability and implementation:** Chimira has been developed as a web application and it is accessible here: http://www.ebi.ac.uk/research/enright/software/chimira.

**Contact:** aje@ebi.ac.uk

**Supplementary information:** Supplementary data are available at *Bioinformatics* online.

## 1 Introduction

Small RNA sequencing data are among the most straightforward types of NGS data to analyse. However many laboratories that generate such data still struggle to develop or adapt computational pipelines for the analysis and interpretation of these data. Additionally, in recent years, it has been reported that many miRNAs go through post-transcriptional alterations that modify their 3′ end, mainly via mono/poly-Uridylation (Heo *et al*., 2009, 2012) or poly-Adenylation (Shanfa *et al.*, 2009). Such modifications are believed to impart significant functional changes to the miRNA. Indeed, other modifications and/or editing have also been observed to occur in several other instances (Burroughs *et al.*, 2010; Sung-Chou *et al.*, 2012; Yu and Chen, 2010). Hence, it is imperative to explore the full profile of all modifications and/or edits that can be identified in small RNA-Seq data. The functional roles of small RNAs in different conditions may be greatly influenced by such modifications. This can be accomplished by aligning small RNA sequences against their hairpin precursors. The alignment region spanning each miRNA is analysed to detect bases in the miRNA sequence that could not possibly have derived from the precursor it aligns to. These unalignable nucleotides are likely either: (i) base-calling errors, (ii) single nucleotide polymorphisms (SNPs), (iii) ADAR edits or (iv) post-transcriptional miRNA modifications (e.g. via TUTases). Base-calling errors are pseudo-random depending on the platform used and usually more likely to occur towards the 3′ end of sequences. In order to study this diverse pool of possible miRNA post-transcriptional modifications, we have developed Chimira. This is a cohesive platform for the processing and analysis of small RNA NGS data allowing simultaneous detection of 3′, 5′ and internal miRNA modifications.

## 2 Input

Chimira accepts FASTQ/FASTA files as input, containing adapter and/or barcode stripped small RNA-Seq data. The user is provided with a simple system for uploading each sample and replicate and selecting among the available run options. Additionally, Chimira provides a limited 3′ adapter cleaning functionality using *reaper* (Davis *et al.*, 2013) supporting different adapters for each input sample. Finally, the system provides a simple interface for computationally determining likely 3′ sequencing adapters in case the user does not have this information.

## 3 Usage notes

In its current release (v1.0), Chimira supports mapping of small RNA-Seq data against 209 genomes registered in miRBase (Griffiths-Jones *et al.*, 2008). In order to optimize and speed-up the analysis, *tally* (Davis *et al.*, 2013) is being used for de-duplicating the sequence fragments. *Tally* dramatically reduces the size of input sequence files by collapsing identical sequences into a single one while storing the total read depth. When input sequence files are uploaded and the appropriate genome is selected, the user is able to launch a new queued job. Every job is submitted to a high-performance computing cluster and the user can follow its progress via an analysis console. Notification via e-mail upon completion of each job is also available, provided the user has supplied a valid e-mail address before launching the job.

## 4 Methods

Two types of miRNAs identification are provided: *Plain Counts* and *Modifications*. *Plain Counts* refers to the quantification of the miRNA molecules that are expressed in any form (template or modified) in each of the input samples. The input sequences are mapped against miRBase using BLASTn (Boratyn *et al.*, 2013) allowing up to two mismatches for each sequence. BLASTn output is filtered so that antisense matches are discarded. The extracted counts are normalized across all samples using DESeq2 (Love *et al.*, 2014) and basic QC plots are provided together with plots for the total counts per sample and the top-10 miRNAs expression levels across all samples. In cases where a small RNA sequence identically matches more than one precursor sequences (i.e. miRNA paralogues) the user can choose between calling only the first optimal alignment hit or assigning counts fractionally with equal weights between the paralogues. Apart from the quantification of miRNA counts and the prevalence of modifications, Chimira integrates basic functionality for comparative analysis of input samples. Specifically, differential expression (of plain counts) between two samples or sets of samples can be visualized through an interactive scatterplot that allows the user to view the miRNA identifier and the different expression levels at each point of the plot (Supplementary Fig. S1). Moreover, the user can display the 10, 20 or 50 most highly expressed miRNAs in two samples (or sets of samples) side by side.

**Fig. 1.**
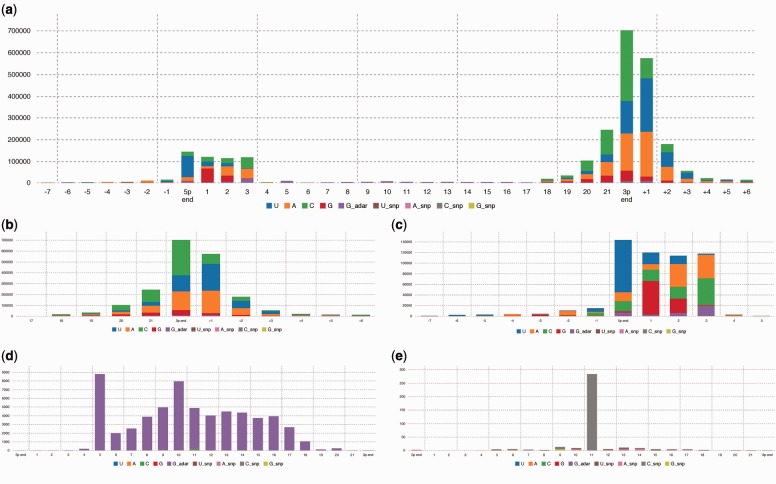
Modification profile from 12 Heart, Liver and Brain tissue samples in *H. sapiens*, as detected by Chimira: (**a**) Global profile (**b**) 3′-Modifications (**c**) 5′-Modifications (**d**) Internal modifications (ADAR edits and SNPs) (**e**) Internal modifications (SNPs). The *x*-axis corresponds to the index positions across a miRNA molecule. They *y*-axis corresponds to the raw counts of the identified modification patterns. The start of a miRNA on the *x*-axis is at index ‘0’ (5′ end) while its end is at index ‘22’ (3′ end)

Secondly, ‘*modifications*’ refers to the quantification of any sequence segments that are part of the input sequences and cannot be explained by genomic sequence. In the example shown (Fig. 1), uridylation and adenylation are the most prevalent modification types in the 1 nt after the 3′ end of the miRNAs, while C modifications are highly enriched exactly at the 3′ end. ADAR editing is the predominant modification type in the internal modifications followed by a moderately expressed C-SNP, 11 nt upstream of the 3′ end (index position: 11).

The types of modifications being identified include:

- **3′-modifications**: any non-templated sequences in a window that starts at the 5th nt upstream of the 3′ end of each miRNA and ends at the 6th nt downstream of the 3′ end.
- **5′-modifications**: any non-templated sequences in a window that starts at the 8th nt upstream of the 5′ end of each miRNA and ends at the 5th nt downstream of the the 5′ end.
- **Internal modifications**: SNPs and ADAR edits. In order for a modification to be classified as a SNP, an arbitrary 70% value is used as a threshold for the ratio of the modified counts to the overall counts.

It is worth noting that the window lengths being used for identification of 3′ and 5′ modifications include nucleotide positions also within the original miRNA sequence to better distinguish all possible modifications from multiple miRNA variants originating from the same precursor but with different length mature products. Modification types are inferred from BLAST alignments of input sequences to their hairpin precursors and then examining the content of alignment mismatches returned. In order to decipher the correct modification position a reference database has been built initially for all supported genomes, containing canonical alignments between mature miRNAs and their hairpin precursors. Based on these data each of the identified modification patterns is assigned a position index (Supplementary Table S1) in order to build the full depth-wise modification profile. Chimira also allows the display of the modification profiles across all samples for a specific miRNA, defined by the user. Finally, all counts (plain and modifications) can be downloaded for further analysis as separate files as soon as the processing of a set of samples is complete.

## 5 Conclusion

Chimira has been specifically developed for fast analysis of simple small RNA datasets with a user-friendly interface. It complements our previous pipeline *sequenceImp*, which was designed for command-line level analysis of larger-scale experiments with complex designs. Chimira is a fast and robust system for the cleaning, filtering, QC and mapping of small RNA-Seq data aiming to simplify the process of small RNA NGS analysis to a straightforward online workflow. Additionally, it allows the extraction of global modification profiles across and within each of the input samples thus allowing the inference of correlations between certain modification patterns and any conditions indicated by the input samples. We hope the system will prove useful to the small RNA community.

*Conflict of Interest*: none declared.

## Supplementary Material

Supplementary Data
