# Supplementary material for: Chimira: analysis of small RNA sequencing data and microRNA modifications
Source: Bioinformatics. 2015 Jun 20;31(20):3365–7. doi: 10.1093/bioinformatics/btv380 (PMC4595902; doi:10.1093/bioinformatics/btv380)
Supplement: Supplementary Data [file supp_31_20_3365__index.html]

Chimira: analysis of small RNA sequencing data and microRNA modifications — Chimira: analysis of small RNA sequencing data and microRNA modifications — Supplementary Data 

# Chimira: analysis of small RNA sequencing data and microRNA modifications

## Supplementary Data

files

- Supplementary Data - pdf file
