# Supplementary material for: Chimira: analysis of small RNA sequencing data and microRNA modifications
Source: Bioinformatics. 2015 Jun 20;31(20):3365–7. doi: 10.1093/bioinformatics/btv380 (PMC4595902; doi:10.1093/bioinformatics/btv380)
Supplement: Supplementary Data [file supp_btv380_Chimira-Supplementary-final_version2.pdf]

Chimira: Analysis of small RNA Sequencing data and microRNA modifications

Supplementary Material

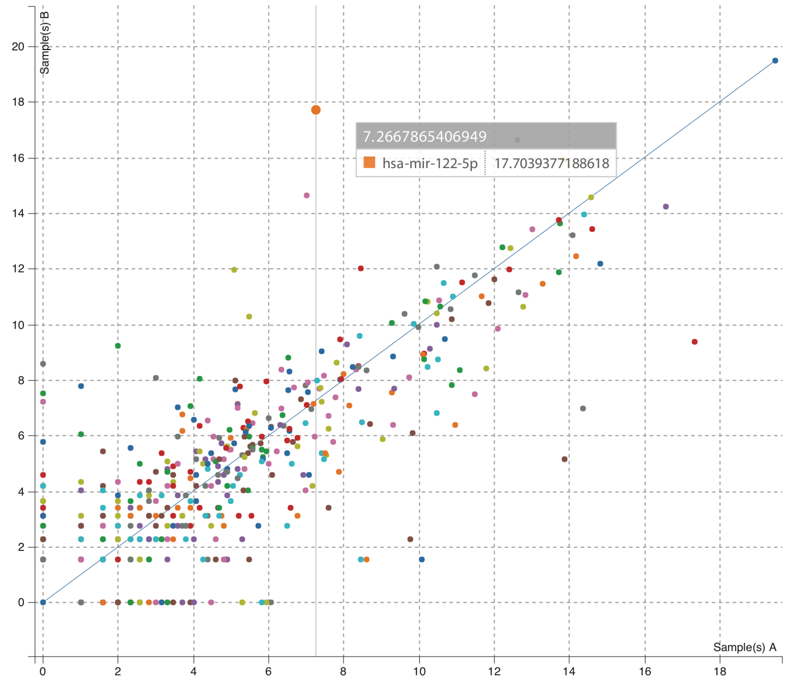

Suppl. Fig. 1. Interactive scatterplot of miRNAs differential expression between a heart and a liver tissue sample. A single miRNA is highlighted showing its identifier (hsa-miR-122-5p) and the $\log_2$ normalised counts in the two samples. As expected, has-miR-122-5p expression is significantly skewed towards the liver sample.

Modification type	Modification position	Description
3'	0	Modification pattern starts from the 3' end of the miRNA
3'	+*k* / -*k*	Modification pattern starts *k* nucleotides downstream / upstream of the 3' end of the miRNA
5'	0	Modification pattern starts from the 5' end of the miRNA
5'	+*k* / -*k*	Modification pattern starts *k* nucleotides upstream / downstream of the 3' end of the miRNA
Internal	0	Modification pattern is precisely at the 5' end of the miRNA
Internal	+*k*	Modification pattern is *k* nucleotides downstream of the 5' end of the miRNA

Suppl. Table 1. Index positions of all modifications relative to the 5'/3' ends of the miRNAs. The directionality of all modification patterns is always considered to be from the 5' to the 3' end.

Infrastructure

All input files are being uploaded and stored into a server using one of the fastest academic networks in Europe. The files content is being validated and an error message is displayed on the user's browser window if it doesn't comply with the allowed input specifications. In any other case, its size is further estimated and based on performance evaluation data acquired form previous training datasets the required resources for submission to a cluster are allocated. After this quality control and pre-processing step, Chimira submits a new queued job to the EMBL-EBI High Performance Computing Cluster. The progress of the whole process can be viewed at all times from an analysis console window that is available at Chimira's progress page, displayed right after a job has been launched. The pipeline Chimira uses for the core analysis is based on Perl (v5.16.0) and R (v3.1.2). A separate thread provided by the Perl API handles each file. Moreover, multiple other threads are launched subsequently during the whole process to initiate cascaded parallel processes for quality control, statistical analysis and merging of various output results, among other tasks. Thread synchronisation and data integrity is coordinated and assured during the whole time of the run. Upon the completion of each job the user is redirected to the results page where he can browse through the output, download all the extracted data (e.g. counts, modifications, etc.) and query the results using the provided interactive tools.

Plain counts analysis

The Plain counts analysis is run by default in either 'Run' or 'Run & Clean' mode. The latter requires trimming of the input files before miRNAs quantification. This is achieved using the reaper utility (Davis et al., 2013) and the adapter sequence or sequences file provided by the user. As soon as an input file is clean from the adapter sequences its reads are uniquified with tally (Davis et al., 2013) and a FASTA file is created recording only one entry for each distinct sequence, accompanied with the respective depth count. This format conversion reduces dramatically the size of the input files and consequently the complexity of the alignment process that follows. The input content is also analysed in order to extract various QC plots (read lengths distribution, nt distribution at each read position and GC content). Input files are then aligned against all miRBase hairpin precursors of the genome that has been selected by the user. Alignment, allowing up to two mismatches, is performed using the Standard Nucleotide Blast (BLASTn, v2.2.24+) and output is being filtered to discard any anti-sense hits. In case the user has selected to split the multi-mapped read counts to their paralogues, the output from BLASTn is being processed in order to identify all paralogues and assign fractionally to them the correct read count values from the multi-mapped hits, using equal weights. If the user has not enabled this option, then only the first BLAST hit is called. Finally, the extracted counts are being post-processed so that they can be visualised and queried in D3.js and C3.js enabled charts and also become downloadable in raw format by the user.

Modification analysis

Modification analysis is also by default part of the 'Run' and 'Run & Clean' mode. This step is performed after the BLAST alignment has been complete. BLAST's output is parsed to identify all mismatches of each hit with the associated subject hairpin precursors. The subject/query start and end indexes are retained and are being used in order to infer the position of the detected mismatches. In order to locate the absolute position of every mismatch across the canonical hairpin precursor sequences, a database has been built for all 209 supported genomes containing the canonical alignments of all mature miRNAs with their respective hairpin precursors, including information about the indexes of the alignment for each case. Based on this information and the depths for each of the detected mismatches, modifications are being assigned a specific modification type and position. Thus, a collection of modifications is assembled eventually containing all identified 3', 5' and internal modifications. SNPs are identified in case the depth of the respective mismatch is found in at least 70% of the associated reads. ADAR edits are called from all A to G transitions provided they occur in at least 90% of all associated sequences. Finally, the extracted modification counts are

being post-processed for visualisation purposes and are bundled in more user-friendly raw formatted files, downloadable by the user.

3' adapter detection feature

In case the 3' adapter associated with a FASTQ file is not known Chimira is able to suggest possible adapter candidates through the 'Other tools' section. This feature is based on the tool minion (Davis et al., 2013) which uses De Bruijn graphs in order to infer potential adapter sequences based on two criteria: a) frequency of occurrence b) attachment to multiple different prefixes. After a list of potential adapters has been created it is being aligned with swan (Davis et al., 2013) against a list of known adapters that we have compiled from various popular sequencing machines and protocols. The best candidate for each input file is selected based on the alignment score of a potential adapter with a known adapter, its length and the end indexes of the alignment.

After an optimized selection process is performed based on the aforementioned criteria a single adapter sequence is suggested for each input file, with a 'Degree of Alignment' score denoting its alignment score with a known adapter sequence. In almost all cases, a suggested adapter with a 'Degree of Alignment' of 100% can be safely used as the correct adapter. This can still be true also for cases where the score is 95% or even less, since the suggested adapter can be just a substring of a known adapter.

In case none of the adapter candidates aligns with any of the verified adapters over a certain threshold ratio (85% by default), a sequence inferred by minion may still be suggested as the adapter of the input samples. However, the Degree of Confidence in that case is 0% and the suggested adapter should be crosschecked manually. This cross-validation might also be needed in case the score is 100% but the length of the suggested adapter is less than ~15nt or the score is e.g. less than 90%. In any case, the trimming efficiency from the use of a particular adapter can be evaluated by inspection of Chimira's stacked modifications profiles and QC plots after a run has been complete.

Comparison of features with other tools

Several other tools are available for performing small RNA-Seq analysis, each providing a different set of features. Some of these tools are web based while others offer stand-alone versions, requiring though many dependencies in some cases. Compared to some of these tools, Chimira does not currently support features such as target or novel miRNA prediction and GO/pathway analysis. As a summary of our feedback experience after trying out each of these tools, we briefly present here the major drawbacks of each tool in comparison with Chimira.

- **UEA workbench** (Stocks et al., 2012)
  Release date: 2012
  Type: stand-alone
  Available genomes from alignment: all miRBase-supported genomes

  UEA workbench is a suite of tools with a rich set of features for small RNA-Seq analysis. Compared to Chimira there is an overhead of installing the application and also some input datasets were falsely recognized to have wrong format when provided as its input. Moreover the analysis cannot be streamlined easily but is rather dependent on setting up locally a working directory tree and dealing manually with the dependencies from each of the available tools. Besides, there are some features that make the use of the tool not very straightforward or flexible. For instance, in order to align small RNA sequences it is also mandatory to provide a long reads file for target prediction at the same time. However, alignment is usually an independent task that its results are used for further statistical or

other RNA analysis, such as target prediction. Finally, UEA does not offer any tool for microRNA modifications extraction from small RNA-Seq data.

- **seqBuster** (Pantano et al., 2010)
  Release date: 2010
  Type: stand-alone
  Available genomes from alignment: Homo sapiens

  seqBuster requires a lot of dependencies and installation is not automated or straightforward. Besides usage instructions are available at a GitHub repository in a non-intuitive format, making the use of this tool to the non-technically savvy rather cumbersome. Apart form this, although seqBuster offers basic 3' adapter removal and tools for miRNAs differential expression it does not provide any utility for the extraction of miRNAs modifications or support for different adapter per input file.

- **OASIS** (Capece et al., 2015)
  Release date: 2014
  Type: web server
  Available genomes from alignment: Homo sapiens, Mus musculus, Drosophila melanogaster, Danio Rerio, Caenorhabditis elegans.

  OASIS does not offer any tools for modifications identification. Besides, the provided tools are not integrated in a very coherent manner since for a single dataset a new job has to be launched in order to perform either sRNA detection, Differential Expression or Classification Analysis. Moreover, no information is provided about the progress of each task except for an e-mail upon reception or completion of each job. The output results are also not being visualized in a queryable manner in contrast with Chimira and only 5 genomes are supported. Finally, upload time and particularly execution time are much higher compared to Chimira's ones.

- **MAGI (**Kim et al., 2014)
  Release date: 2013
  Type: web server
  Available genomes from alignment: Homo sapiens

  MAGI web server allows the alignment against only the human genome. Although it uses web workers for downsizing the input files before upload, total upload time is not significantly lower than for Chimira when analyzing the same dataset in these two web servers. Besides, input files need to follow a specific naming scheme (with group annotation) and have to be de-compressed before upload, which is very impractical for large datasets in terms of local disk space requirements. In this case again, no modifications information is extracted and the user cannot query the results interactively.

There are only two other already published tools that offer some type of modifications detection. Their performance and functionality are briefly discussed below:

- **CAP-miRSeq (**Sun et al., 2014)
  Release date: 2015
  Type: stand-alone
  Available genomes from alignment: manually installed from the user

Although it requires the installation of a virtual machine and import of the developed Linux virtual environment, the whole setup is easy and straightforward. However, applying any of the provided tools involves an extra overhead of downloading the genome and/or annotation files and creating manually the configuration files. Besides, with regards to the modifications identification, CAP-miRSeq only allows the detection of single nucleotide variants (SNV) and does not support 3'/5' modifications or ADAR edits detection.

- **CPSS (**Zhang et al., 2012)
  Release date: 2010
  Type: web server
  Available genomes from alignment: Homo sapiens, Mus musculus, Rattus norvegicus, Pan troglodytes, Gallus gallus, Bos taurus, Canis lupus familiaris, Pongo abelii, Sus scrofa, Danio rerio

  The CPSS web server allows miRNA isoforms detection, supporting 3', 5' modifications and SNPs. However, the extracted results do not directly provide the modifications information in a fully quantitative format. Specifically, all detected isoforms are displayed just as an aligned stack of sequences and a summary table is provided only for the total number of modifications in either of the 3' or 5' ends with no extra information about their content or exact positions. Besides, no ADAR edits detection is supported. Apart from that, CPSS's interface allows upload of only one file at a time. Upload speed is very low (50 MBs upload requires over 40 min on average) and even though a script is provided for pre-trimming/cleaning of the input files the post-processed files' size is not significantly reduced. Thus, the web server is not practically usable and as a result it has not been included in the Benchmarking tests.

Benchmark

We are providing here the benchmarking results from the comparison of Chimira with other already published web servers. We tested two different aspects from each application: a) upload time and b) execution time, using three different datasets of increasing input size. Total upload and execution times are provided in **Suppl. Tables 2** and **3** (raw data) and displayed in **Suppl. Fig. 2**.

	**Total execution time (min)**		
**Compressed (gzip) Input Size in GB**	**Chimira**	**OASIS**	**MAGI**
0.3	4.5	65	58
2.7	24	103	92
9.3	43	171	1650

Suppl. Table 2. Execution times (raw data) of Chimira, MAGI and OASIS methods for three different input datasets of small, medium and large size.

	**Upload time (min)**		
**Compressed (gzip) Input Size in GB**	**Chimira**	**OASIS**	**MAGI**
0.3	0.83	0.92	0.68
2.7	4.5	8.5	4
9.3	19	26	16

Suppl. Table 3. Upload times (raw data) of Chimira, MAGI and OASIS methods for three different input datasets of small, medium and large size.

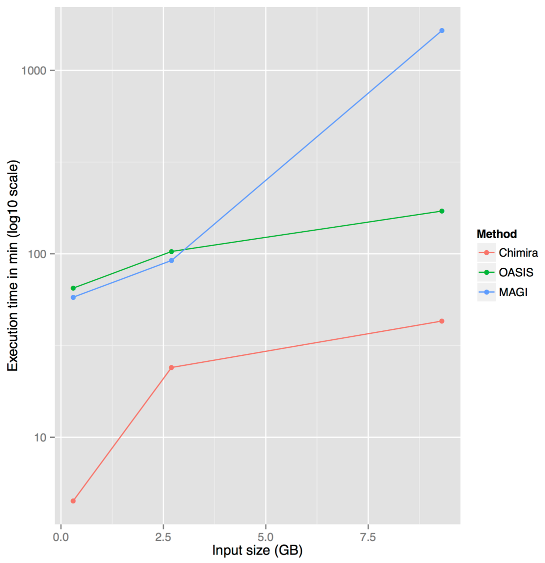

a.

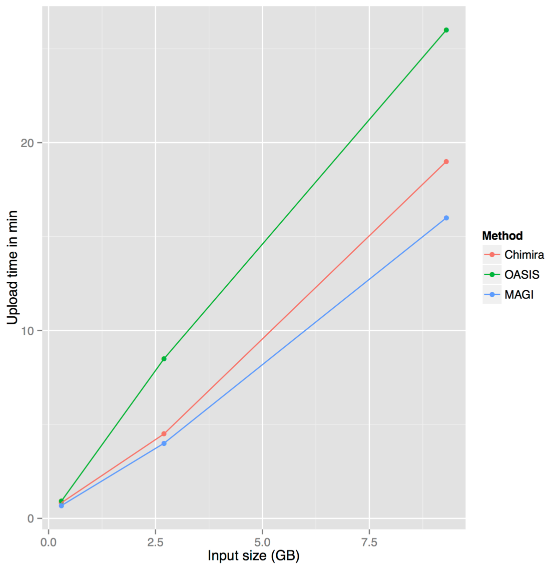

b.

Suppl. Fig. 2. Scatterplots with: a) execution and b) upload times of Chimira, MAGI and OASIS methods for three different input datasets of small, medium and large size. Y-axis in sub-figure (a) has been normalized to a log10 scale.

Based on the benchmarking plots above we notice that Chimira's upload times are almost identical with MAGI's ones, which are the lowest. With regards to execution time, Chimira is evidently outperforming any of the other two tools for all input size ranges.

Validation of Chimira's results with already published work.

a) ADAR editing

We present here a comparison of Chimira's results with the results obtained by an already published study regarding ADAR editing events. This study examined the effect of ADAR2 knockout on A to I editing and abundance of mature miRNAs (Vesely et al., 2014). A brief list of points for validation of Chimira's results follows:

- The study reported mmu-miR-378a as the most abundant miRNA and no significant change in the abundance of the 5 most abundant miRNAs, which also make up for the 48% of all identified miRNAs.

  Chimira's respective results are presented in Suppl. Tables 4-5 and Suppl. Fig. 3.

miRNA	DESeq2 Normalised Counts
mmu-mir-378a-3p	21352261
mmu-mir-9-5p	14282621
mmu-mir-127-3p	7922293
mmu-mir-183-5p	6927689
mmu-mir-182-5p	6735387

Suppl. Table 4. Top five most abundant miRNAs, as identified by Chimira.

	Chimira	Vesely et al., 2014
Total **depth**	126208757	-
5 most abundant miRNAs **depth**	57220251	-
5 most abundant miRNAs **ratio**	45.3 %	48 %

Suppl. Table 5. Comparison of the top five most abundant miRNAs depth ratios.

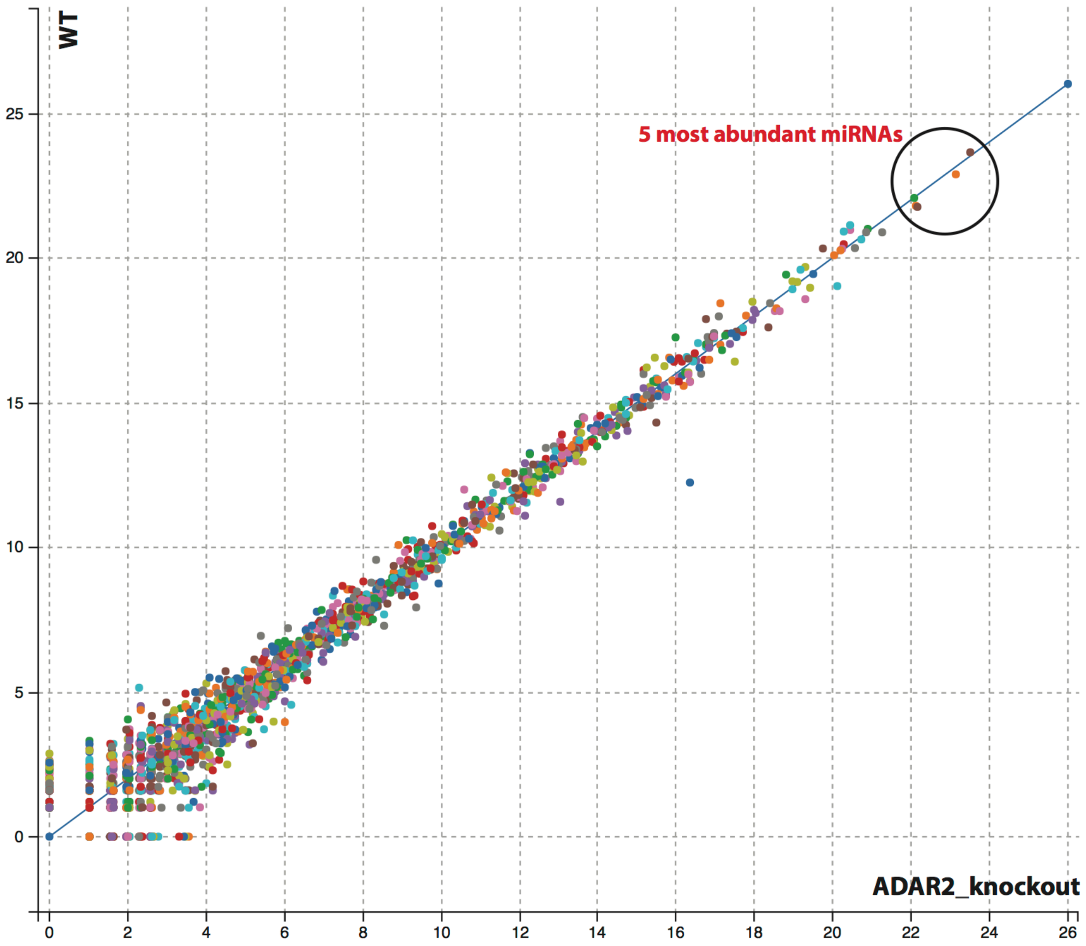

Suppl. Figure 3. Differential expression of all miRNAs between WT and ADAR2-knockout samples, as returned by Chimira. The expression of the 5 most abundant miRNAs doesn't change significantly between the two conditions.

Based on the data presented above we observe that Chimira's output results are complying very well with the examined study's results.

- As expected, the study reported an overall decrease in ADAR editing events in the ADAR2 knockout samples. Chimira is capturing this global ADAR editing dropout through the global modifications profiling in all samples, as shown in Suppl. Fig. 4.

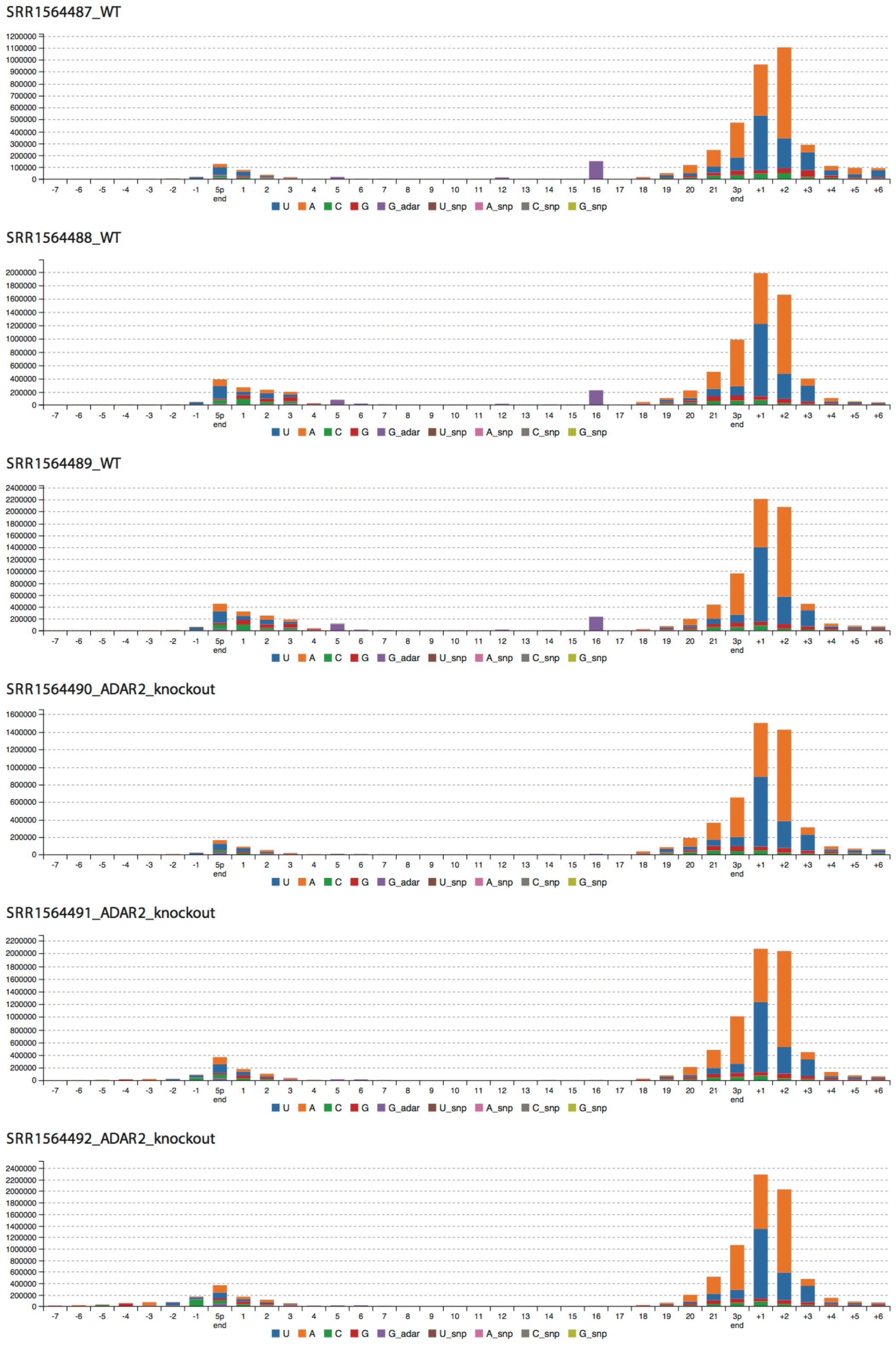

Suppl. Fig. 4. Visualisation of overall dropout of ADAR editing events (purple colour) in the ADAR knockout samples compared to the WT ones.

- Finally, Chimira is also identifying successfully the ADAR edit events for individual miRNAs. We compare in Suppl. Table 6 the results of the examined study with Chimira's output for a representative list of 5 miRNAs mentioned in that paper (Vesely et al., Table 4).

	Chimira			Vesely et al., 2014		
**miRNA**	*Position*	*% editing (WT)*	*% editing (Knockout)*	*Position*	*% editing (WT)*	*% editing (Knockout)*
mmu-mir-378a-3p	16	6.08	0.12	16	6.3	0.2
mmu-mir-379-5p	5	44.5	10.8	5	46.4	11.7
mmu-let-7e-5p	19	28.3	41.2	19	29.1	42.7
mmu-mir-3099-3p	7	87	80.7	7	79.8	66.1
mmu-mir-421-3p	14	14.3	3.6	14	10.9	3.3

Suppl. Table 6. Comparison of significant editing events in a list of 5 miRNAs (detected in all three replicates).

We can notice that the results returned by Chimira are very similar with the ones returned by Vesely et al.'s study.

All Chimira's output for this particular dataset is provided for reference at the following link:
http://wwwdev.ebi.ac.uk/enright-srv/chimira/cgi-bin/core/display_results.php?uuid=a46439f8-e5ca-08be-f4dc-60ef440f731d&jobs_num=6

b) TUT4/7 knockout

We present here Chimira's performance in efficiently detecting drop in uridylation levels in TUT4/7 knockout samples. For this type of validation, we have used a dataset with one WT and one TUT4/7 knockout sample (Liu et al., 2014).

We show in Suppl. Fig. 5 that overall uridylation level drops significantly in the knockout samples (~8-fold decrease).

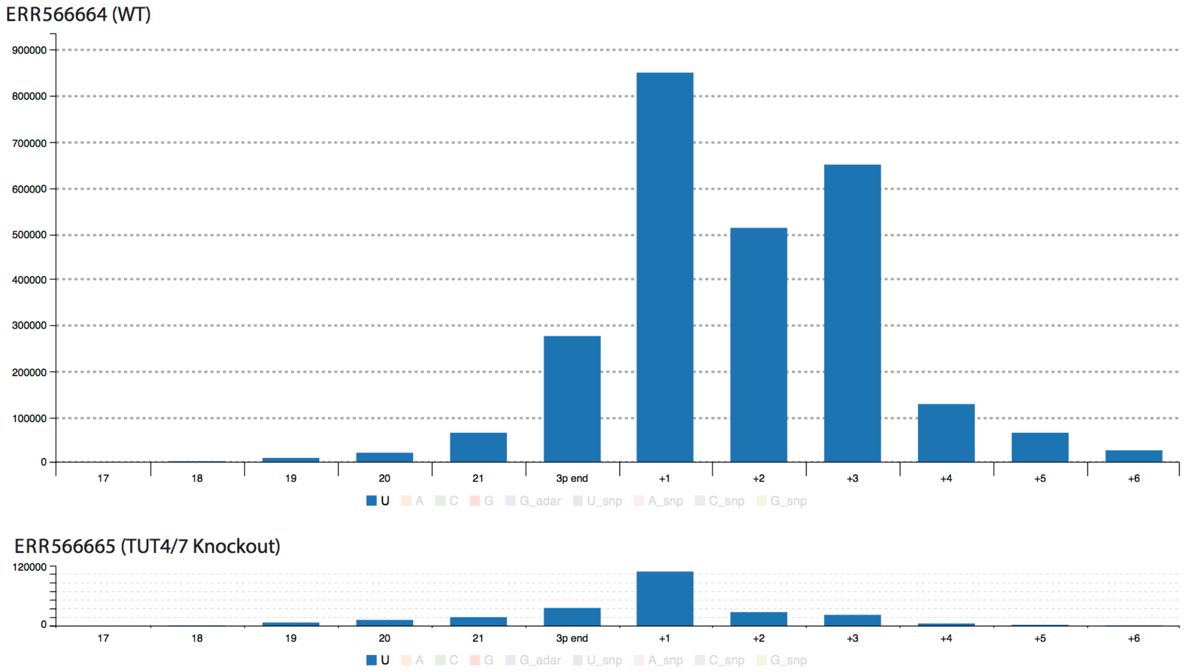

Suppl. Figure 5. Uridylation levels in the WT and TUT4/7 samples of Liu et al.'s study (accession number: PRJEB6759), as extracted by Chimira (both plots are on the same scale).

After processing Chimira's output plain and modification counts we have plotted the change in uridylation levels between the WT and Knockout samples as well as the overall expression of all the uridylated miRNAs in the two samples. Similarly with the points presented in the examined study, we can see in Suppl. Fig. 6 that uridylation levels, for each miRNA individually, are skewed towards the WT sample, which has already been shown in Suppl. Fig. 5 from a global perspective. Besides, in Suppl. Fig. 7 we can see that some miRNAs are expressed more highly in the knockout samples.

This observation is in accordance with the results of the examined paper that certain miRNAs where up-regulated in the Knockout sample, which can be explained by faster degradation of the mature miRNA products due to uridylation induced by the TUT4 and TUT7 enzymes.

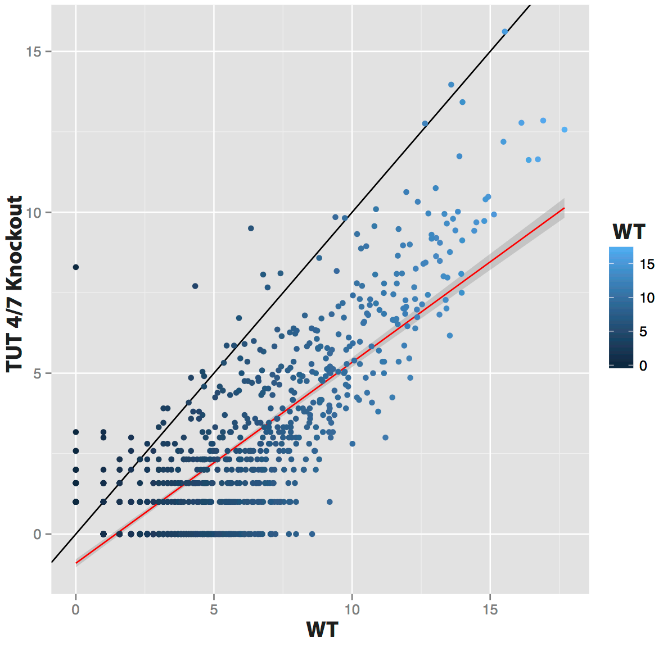

Suppl. Figure 6. miRNAs uridylation levels across the WT and TUT4/7 Knockout samples (log2 of DESeq2 normalised counts).

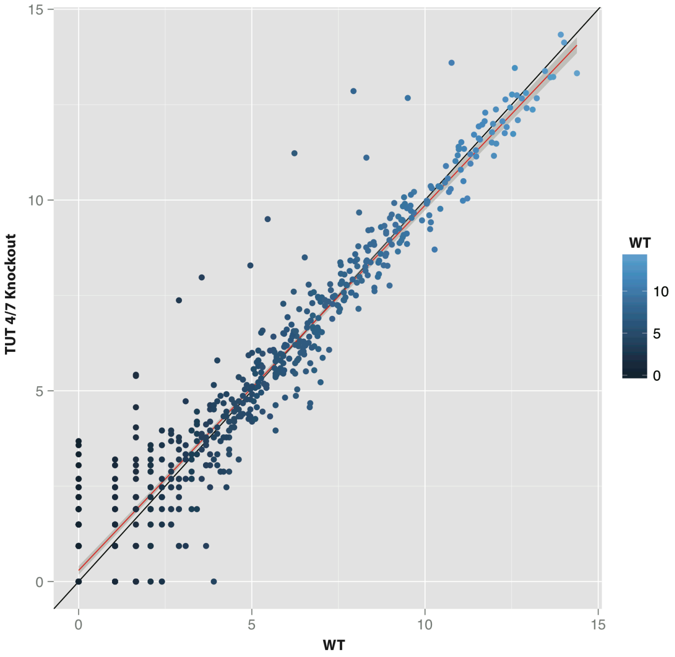

Suppl. Figure 7. Uirdylated miRNAs overall expression across WT and TUT4/7 Knockout samples (log2 of DESeq2 normalised counts).

All Chimira's output for this particular dataset is provided for reference at the following link:
http://wwwdev.ebi.ac.uk/enright-srv/chimira/cgi-bin/core/display_results.php?uuid=f1a4e091-66c8-17b8-9d57-6be8f8f92ead&jobs_num=2

Tools/Resources versions

Tool / Resource	Version
**miRBase**	Release 21
**BLASTn**	2.2.24+
**Reaper**	15-065
**Minion**	15-065
**R**	3.1.2
**Perl**	5.16.0
**Perl CGI**	3.59
**PHP**	5.3.3
**JavaScript** (recommended)	>= 1.7
**Fine-uploader** (JavaScript plugin)	5.1.3
**jQuery**	1.10.2
**jQuery UI**	1.11.4
**D3.js**	3.5.0
**C3.js**	0.4.8
**DataTables** (jQuery plugin)	1.10.0

Chimira is supported by all popular web browsers (e.g. Chrome, Safari, Firefox) that run on personal computers and is also accessible by any JavaScript enabled browser on mobile devices.

Acknowledgements

We gratefully thank Matthew P.A. Davis and Stijn van Dongen for comments on our manuscript.

Supplemental References

Capece et al. (2015). Oasis: online analysis of small RNA deep sequencing data. *Bioinformatics*, pii: btv113v2.

Kim et al. (2014). MAGI: a Node.js web service for fast MicroRNA-Seq Analysis in a GPU Infrastructure. *Bioinformatics*, 30, 2826-2827.

Liu et al. (2014). A MicroRNA Precursor Surveillance System in Quality Control of MicroRNA Synthesis. *Molecular Cell*, 55, 1-12.

Pantano, L. et al. (2010). SeqBuster, a bioinformatic tool for the processing and analysis of small RNAs datasets, reveals ubiquitous miRNA modifications in human embryonic cells. *Nucleic Acids Research*, Vol. 38, No. 5, e34.

Stocks, M. B. et al. (2012). The UEA sRNA workbench: a suite of tools for analysing and visualizing next generation sequencing microRNA and small RNA datasets. *Bioinformatics*, 28, 2059-2061.

Sun et al. (2014). CAP-miRSeq: a comprehensive analysis pipeline for microRNA sequencing data, *BMC Genomics,* 15:423.

Vesely et al. (2014). ADAR2 induces reproducible changes in sequence and abundance of mature microRNAs in the mouse brain. *Nucleic Acids Research*, Vol. 42, No. 19, 12155-12168.

Zhang et al. (2012). CPSS: a computational platform for the analysis of small RNA deep sequencing data. *Bioinformatics,* 28, 1925–1927.
